# Molecular and cellular mechanisms of tight junction dysfunction in the irritable bowel syndrome

**DOI:** 10.3892/mmr.2015.3808

**Published:** 2015-05-21

**Authors:** PENG CHENG, JIANNING YAO, CHUNFENG WANG, LIANFENG ZHANG, WUMING KONG

**Affiliations:** 1 Department of Gastroenterology, First Hospital of Zhengzhou University, Zhengzhou, Henan 450052, P.R. China; 2 Department of Gastroenterology, Affiliated Sixth People's Hospital of Shanghai Jiaotong University, Shanghai 201306, P.R. China

**Keywords:** irritable bowel syndrome, tight junctions, claudin-1

## Abstract

The pathophysiological mechanisms of the irritable bowel syndrome (IBS), one of the most prevalent gastrointestinal disorders, are complex and have not been fully elucidated. The present study aimed to investigate the molecular and cellular mechanisms of tight junction (TJ) dysfunction in IBS. Intestinal tissues of IBS and non-IBS patients were examined to observe cellular changes by cell chemical tracer electron microscopy and transmission electron microscopy, and intestinal claudin-1 protein was detected by immunohistochemistry, western blot analysis and fluorescence quantitative polymerase chain reaction. Compared with the control group, TJ broadening and the tracer extravasation phenomenon were observed in the diarrhea-predominant IBS group, and a greater number of neuroendocrine cells and mast cells filled with high-density particles in the endocrine package pulp as well as a certain extent of vacuolization were present. The expression of claudin-1 in diarrhea-predominant IBS patients was decreased, while it was increased in constipation-predominant IBS patients. In conclusion, the results of the present study indicated that changes in cellular structure and claudin-1 levels were associated with Tjs in IBS.

## Introduction

As a crucial component of gastrointestinal homeostasis, the intestinal epithelial barrier is the first line of defense against numerous adverse factors, including toxins, certain antigens and pathogenic microorganisms. Dysfunction and destruction of molecules and functional proteins of the intestinal epithelial barrier results in the disturbance of the latter, leading to activation of mucosal immune responses, which are associated with the pathogenesis of intestinal disorders. Among these, irritable bowel syndrome (IBS) is one of the most common chronic, relapsing-remitting inflammatory diseases (1,2).

The pathophysiological mechanisms of IBS are complex and have not been fully elucidated; however, they are reckoned to be multifactorial. Of note, the dysregulation of the brain-gut axis and cross-regulation between the central and the enteric nervous system has attracted attention in recent studies (3). Three essential factors are required in the pathogenesis of IBS: Breakdown of intestinal barrier function, activation of lamina propria immune cells by luminal contents and, importantly, an overexaggerated immune response (4).

Tight junctions (TJ) are apical-host adhesive junctional complexes in epithelial cells, and regulate proliferation, polarization and differentiation of mammalian gut cells (5,6). Between the apical and lateral membrane, a continuous belt-like ring is formed by TJs around epithelial cells, which regulates the selective/semipermeable transportation of paracellular ionic solutes and intercellular signaling responses (7,8). The biological importance of TJs was initially recognized in the 1960s with the emergence of electron microscopy (9). TJs are a series of multiprotein complexes of dynamic function, of which claudins, occludin, junctional adhesion molecules (JAMs) and tricellulin are four unique families of transmembrane proteins (9).

According to recent studies, claudins are 20- to 27-kDa integral membrane proteins and consist of two extracellular loops, four hydrophobic transmembrane domains as well as N- and C-terminal cytoplasmic domains (7,10–12), among which two extracellular loops are essential for their homophilic and heterophilic properties. Moreover, interactions of TJ proteins form ion-selective channels, facilitating the passage of iron and selective solutes from the intercellular space and preventing adverse events caused by toxins or microorganisms (7).

The regulatory role of claudins in barrier function has been proved in recent studies on claudin-deficient mice (12), and Furuse *et al* (13) found that claudin1^−/−^ mice suffered significant transepidermal water loss and died within the first day following birth. The present study aimed to investigate the molecular and cellular mechanisms of TJ dysfunction in IBS, particularly the role of claudin1.

## Materials and methods

### Patients

Bowel tissues from 93 IBS patients were collected at the First Hospital of Zhengzhou University (Zhengzhou, China). The patients consisted of 58 women and 35 men, and they were diagnosed according to the Rome III criteria (14). Electron microscopic observation was performed on specimens from 10 patients with diarrhea-predominant IBS (mean age, 48.5 years; range, 19–68 years) and 10 patients with constipation-predominant IBS (mean age, 46.3 years; range, 18–65 years) and bowel tissues from 10 patients unaffected by IBS with bleeding hemorrhoids (mean age, 50.2 years; range, 17–71 years). Furthermore, claudin-1 was investigated in specimens from 23 patients with diarrhea-predominant IBS (mean age, 39.7 years; range, 18–65 years) and 20 patients with constipation-predominant IBS (mean age, 38.6 years; range, 19–70 years) and bowel tissues from 20 patients unaffected by IBS with bleeding hemorrhoids (mean age, 40.1 years; range, 17–71 years).

### Specimens

All selected patients were separately subjected to electronic colonoscopy, and four biopsies were taken from the terminal ileum and ascending colon mucosa, respectively. Specimens were at least 0.2×0.2×0.2 cm in size. The use of human tissue was approved by the Ethics Committee of the First Hospital of Zhengzhou University (Zhengzhou, China) and all patients gave informed consent.

### Reagents and antibodies

Rabbit anti-claudin-1 (cat. no. 50011919) was purchased from Zymed Laboratories. Goat anti-rabbit immunoglobulin (Ig)G antibody labeled with horseradish peroxidase (HRP) (cat. no. ZB-2301) was purchased from Zhongshan Golden Bridge Biotechnology Company (Beijing, China). Taurocholic acid (TCA) and glycocholic acid (GCA) were purchased from Amresco LLC (Solon, OH, USA), and deoxycholic acid (DCA) was purchased from Invitrogen Life Technologies (Carlsbad, CA, USA). β-actin antibody (cat. no. A5441) was purchased from Sigma-Aldrich (St. Louis, MO, USA).

### Specimen preparation and ultrastructure observation of intestinal TJs

Electron microscopy (V6458; Pentax, Tokyo, Japan) using cytochemical techniques with a lanthanum nitrate tracer was used to evaluate the samples. Sample specimens were cut into 1-mm^3^ squares and marked regarding their orientation. Tissue samples were fixed in phosphate-buffered saline (PBS; Xi'an Chemical Reagent Company, Xi'an, China) containing 3% glutaraldehyde (Xi'an Chemical Reagent Company), 1.5% (0.1 mol/l) paraformaldehyde (Xi'an Chemical Reagent Company) and 1% lanthanum nitrate (pH 7.2; Xi'an Chemical Reagent Company) at 4°C for 2 h, then immersed in 0.1 mol/l sodium cacodylate buffer for 30 min, fixed with 1% osmium tetroxide fixative (containing 1% lanthanum nitrate; pH 7.2; Xi'an Chemical Reagent Company) at 4°C for 1.5 h, washed with 0.1 mol/l sodium cacodylate buffer (Xi'an Chemical Reagent Company) for 1 min and dehydrated with a graded series of ethanol (Xi'an Chemical Reagent Company): 30% ethanol for 5 min, 50% ethanol for 5 min, 70% ethanol for 5 min, 90% ethanol 5 min twice and pure ethanol for 5 min three times. Samples were then dehydrated with acetone for 5 min and embedded with Epon618 epoxy resin (Xi'an Chemical Reagent Company). The embedded tissues was cut into 90-nm slices. Finally, TJs were observed by using a Hitachi H-600 projection electron microscope (Hitachi, Tokyo, Japan) and images were captured.

### Specimen preparation and ultrastructural observation of intestinal epithelial cells

Samples were cut into 1-mm^3^ small squares and immediately placed in 0.1 M PBS containing 2.5% glutaraldehyde and 4% paraformaldehyde at 4°C for 2 h, then immersed in 0.1 M PBS for 30 min, fixed with 1% osmium tetroxide in 0.1 M PBS at 4°C for 2 h, washed by 0.1 M PBS for 1 min, and then dehydrated in a graded series of ethanol: 30% ethanol for 10 min, 50% ethanol for 10 min, 70% ethanol for 10 min, and 70% ethanol; 90% ethanol 10 min twice and pure ethanol for 10 min three times. Samples were the incubated in propylene oxide for 10 min, then dehydrated with acetone for 5 min and embedded in Epon812 epoxy resin. After polymerization, half ultrathin sections of 1–2 *µ*m were cut and positioned under a light microscope after methylene blue staining (Xi'an Chemical Reagent Company) for 30 sec at room temperature. Ultrathin sections of 50-70 nm were cut using an LKB-V ultramicrotome machine (LKB-8800; Lanka Electricity Company, Bromma, Sweden). After uranyl acetate and lead citrate staining, tissues were observed using the Hitachi H-600 projection electron microscope and images were captured.

### Fluorescence quantitative polymerase chain reaction (FQ-PCR) analysis of intestinal claudin-1 mRNA expression

#### Extraction of total RNA samples

The tissue sample was placed in a glass of homogenizer, TRIzol (50 mg/ml) was added according to the manufacturer's instructions, and tissue was homogenized over 5 min. Following centrifugation at 17,000 x g for 5 min at 4°C, the pellet was discarded. Chloroform was added at a ratio of 1:5 with regard to TRIzol followed by ultrasonication for 15 min at room temperature. Following centrifugation at 17,000 x g for 10 min, the supernatant was discarded by adding 1 ml water and transferring the sample to a fresh centrifuge tube. Isopropanol was added at a ratio of 1:2 with regard to TRIzol followed by incubation for 10 min at room temperature. The supernatant was discarded after centrifugation at 4°C and 9,000 x g for 5 min. 1 ml 75% ethanol was then added to suspend the precipitate, followed by centrifugation at 4°C and 9,000 x g for 5 min, and the supernatant was discarded. 20 *µ*l diethylpyrocarbonate (DEPC)-treated water was added to dissolve the precipitate, followed by incubation at 60°C for 5 min. The optical density (OD) value was measured using a DNM-9606 Microplate reader (PuLang, Nanjing, China) to determine the concentration and purity of the mRNA.

#### Synthesis of cDNA

Oligo (dT) 18 primer (Huada, Shenzhen, China) (1 *µ*l) was added to 0.5 *µ*g template RNA, and DEPC-treated distilled water (Xi'an Chemical Reagent Company) to a final volume of 12 *µ*l. The mixture was gently agitated and centrifuged at 17,000 × g for 3–5 sec. The mixture was incubated at 70°C for 5 min, cooled on ice and 5X reaction buffer (4 *µ*l), RiboLock™ ribonuclease inhibitor (20 U/*µ*l; 1 *µ*l), 10 mM deoxyribonucleotide triphosphate mix (2 *µ*l) were added (all from Thermo Fisher Scientific, Pittsburgh, PA, USA), followed by incubation at 37°C for 5 min. Subsequently 1 *µ*l RevertAid™ Moloney murine leukemia virus reverse phase transcriptase (200 U/*µ*l; Thermo Fisher Scientific) was added, followed by incubation at 42°C for 60 min. Following incubation at 70°C for 10 min, the reaction was terminated by rapidly placing the sample on ice, followed by preservation at −20°C.

#### FQ-PCR detection

According to the target gene in GenBank (www.ncbi.nlm.nih.gov/genbank/), primers were designed using Oligo 6.64 software (Molecular Biology Insights, Inc., Colorado Springs, CO, USA), and are shown in Table I. A 25-*µ*l reaction system containing SYBR Primix (12.5 *µ*l), ROX deference dye (0.5 *µ*l), upstream and downstream primer (20 pmol, respectively) and template cDNA (50 ng) was used (all from Invitrogen Life Technologies). A real time PCR instrument was used with a three-step PCR reaction program (ABI 7000; Applied Biosystems, Life Technologies, Thermo Fisher Scientific, Waltham, MA, USA). The reaction conditions were as follows: Denaturation at 50°C for 2 min and 95°C for 2 min, 1 cycle; denaturation at 95°C for 15 sec, annealing at 55°C for 30 sec, extension at 72°C for 30 sec, over 45 cycles. The dissolution profile of the PCR reaction solution was determined for each sample. The CT value of each sample was calculated using ABI prime 7000 SDS software (Applied Biosystems), and the relative gene expression in each group was calculated according to the 2^−ΔΔCT^ method, with ΔΔCT=(CT_Target_−CT_GAPDH_)_Time x_−(CT_Target_−CT_GAPDH_)_Time 0_, where Time x represented the expression of the target gene at the time point of interest and Time 0 represented the target gene 1-fold expression normalized to GAPDH.

#### Western blot analysis of intestinal claudin-1 protein

Total intestinal protein extracted using the radioimmunoprecipitation assay (RIPA) total protein extraction kit (990 *µ*l RIPA buffer and 10 *µ*l phenylmethanesulfonylfluoride; Amresco). For blotting, 10% separating gel (10 m) and 5% stacking gel (5 ml) (Amresco) were used. Sample and loading buffer were mixed at a ratio of 4:1, heated for denaturation at 100°C for 5 min, and 15 *µ*l were loaded onto the gel following instantaneous centrifugation. Proteins were separated using 10% SDS-PAGE at 80 V for 90 min, until bromophenol blue (Amresco) reached the bottom of the resolving gel. The gel was then immersed with Coomassie blue dye (Amresco) and the blot was transferred onto a polyvinylidene difluoride (PVDF; Amresco) membrane at 25 V for 2.5 h. the PVDF membrane was stained with Ponceau S (Amresco) for 5 min. The marker position was located using India Ink (Amresco). The membrane was washed with distilled water several times until completely clear and then blocked with 8% skimmed milk. Following agitation in Tris-buffered saline with Tween 20 (TBST; Sigma-Aldrich) for 60 min, the membrane was rinsed with TBST three times. Then antibody (rabbit anti-human claudin-1 polyclonal antibody at a dilution of 1:120) was added, followed by incubation with agitation at 37°C for 2 h and subsequent incubation at 4°C overnight. The membrane was rinsed with TBST three times, followed by addition of the secondary antibody [goat anti-rabbit IgG-HRP conjugate at a dilution of 1:500] and gentle agitation for 3 h. The membrane was rinsed in TBST three times for a total of 30 min, and an enhanced chemiluminescence kit (BioTeke Corporation, Beijing, China) was used for antibody visualization. The blots were exposed to X-ray film (Kodak, Tokyo, Japan) and a gel imaging acquisition system (HQ-320XT Film Washing Machine; Huqiu Imaging Technologies (Suzhou) Co., Ltd., Suzhou, China) was applied to analyze the results.

#### Immunohistochemical analysis of intestinal claudin-1 protein

The tissue sections were dewaxed and hydrated with xylene and graded alcohol, then were incubated with 3% H_2_O_2_ for 10 min. The tissue sections were fixed with citrate buffer in the microwave (high-power for 3 min, and low-power for 10 min; Galanz Enterprise Group Co., Ltd., Guangdong, China). Primary antibody (rabbit anti-human claudin-1 polyclonal antibody; 1:150 dilution) was added followed by incubation at 4°C overnight. Samples were washed with PBS three times for 3 min each. Goat anti-rabbit-HRP IgG was added as the secondary antibody, followed by incubation at 37°C for 30 min. Samples were washed with PBS three times for 3 min each and antibodies were visualized with diaminobenzidine. Samples were washed with distilled water, stained with hematoxylin, dehydrated with alcohol, washed with xylene, sealed with flavor sealing tablets and images were captured using a microscope (IX50; Olympus, Tokyo, Japan). Images were quantitatively evaluated using Image Pro Plus software, version 5.1 (Media Cybernetics, Rockville, MD, USA).

#### Statistical analysis

All values are presented as the mean ± standard error and all statistical analysis was conducted using SPSS software, version 19.0 (IBM SPSS, Armonk, NY, USA). Data were analyzed using Student's t-test for two independent groups or one-way analysis of variance followed by Fisher's paired least-significant difference or Scheffe's F test for multiple comparisons. P<0.05 was considered to indicate a statistically significant difference between values.

## Results

### Ultrastructural observation of intestinal TJs

**Figure 1:**
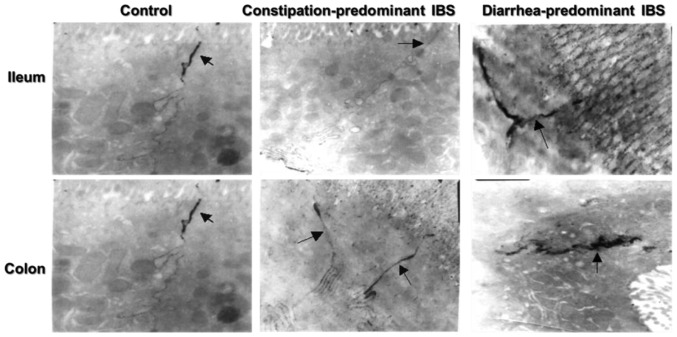
Ultrastructural observation of intestinal TJs (indicated by arrows) using electron microscopy (magnification, ×20,000). Intercellular TJ structures of the terminal ileum and ascending colon mucosa of constipation-predominant IBS was as normal as that of the control group, and no widened gaps and no tracer extravasation phenomenon were observed. In diarrhea-predominant IBS, TJ gaps of the ileum mucosa and ascending colonic mucosa were widened, and varying degrees of tracer extravasation phenomenon were observed. TJ, tight junction; IBS, irritable bowel syndrome.

Electron microscopic observation showed that the terminal ileum and ascending colon mucosa of constipation-predominant IBS was as normal as that of the control group (Fig. 1); intercellular TJ structures were not widened and no tracer extravasation phenomenon was observed. Among the 10 diarrhea-predominant IBS cases, gaps between TJs were widened in the ileum mucosa in seven cases and in the ascending colonic mucosa in eight cases, and varying degrees of tracer extravasation phenomenon were observed. Among four patients with a history of infectious diarrhea in addition to IBS, the terminal ileum and ascending colon mucosa of three cases displayed these changes.

### Ultrastructural observation of intestinal epithelial cells

**Figure 2:**
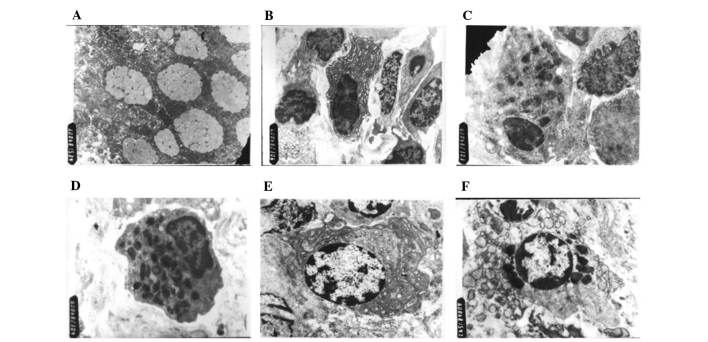
(A) Colon and small intestinal epithelium, an increased mucus secretion and mucus bubble fusion was observed in goblet cells which were in a secreted exuberant state. (B) Plasma cells, the rough endoplasmic reticulum was dilated and mitochondria increased significantly. (C) Normal epithelium of the small intestine and colon mucosa, a small quantity of neuroendocrine cells were observed. The endocrine particles were fewer and no significant alterations in the vacuoles were observed. (D) Neuroendocrine cells in the two types of IBS patients, cells were demonstrated to be cone-shaped and large in the upper end and small in the other, with nuclei at the upper end and the cytoplasm at the lower end. The cytoplasm of neuroendocrine cells was filled with a high density of endocrine granules, several of which were vacuole-like. (E) Mast cells of normal colonic mucosa, a low density of particles was present and the small intestine had no vacuoles. (F) The mast cell cytoplasm in the two types of IBS patients, a high density of particle vacuoles were present in the cytoplasm, which were in the active degranulation state. IBS irritable bowel syndrome.

Electron microscopic observation (Fig. 2) showed that compared with the control group, the diarrhea-predominant IBS and constipation-predominant IBS groups displayed significantly increased mucus secretion and mucus bubble fusion in goblet cells of the colon and small intestinal epithelium, which were in a state of exuberant secretion (Fig. 2A). Plasma cells of the above mentioned four IBS patients with a history of infectious diarrhea were increased, of which the rough endoplasmic reticula were dilated and mitochondria were significantly increased (Fig. 2B). The structural changes of constipation-predominant IBS were not manifested. Neuroendocrine cells were present in the two types of IBS patient, which were cone-shaped, big in the upper end and small in the other, with nuclei located at the upper end and the cytoplasm at the lower end. The cytoplasm of neuroendocrine cells was filled with a high density of endocrine granules, several of which were vacuole-like, while in the normal control samples, fewer neuroendocrine cells were observed in the small intestine and colon mucosa, the endocrine particles of which were fewer, and no significant changes in vacuoles were present (Fig. 2C and D). In addition, certain vacuoles were fully filled with high-density particles in the mast cell cytoplasm in the two types of IBS patients, which were in the active degranulation state, while a low density of particles and no vacuoles were observed in the mast cells of the colonic mucosa and small intestine of the healthy control samples (Fig. 2E).

### Claudin-1 is upregulated in constipation-predominant IBS and downregulated in diarrhea-predominant IBS

**Figure 3:**
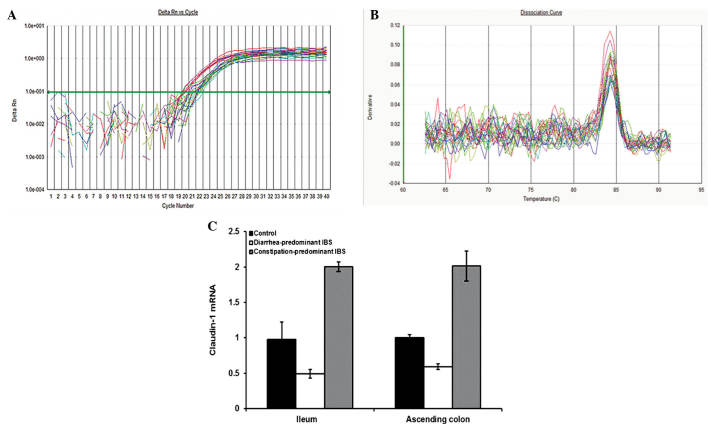
FQ-PCR analysis of intestinal claudin-1 mRNA expression. (A) FQ-PCR amplification curves of claudin-1. (B) FQ-PCR dissolution curves of claudin-1. (C) Quantified results showed that compared with claudin-1 mRNA expression in the small intestine of the control group, that in diarrhea-predominant IBS was significantly decreased (P<0.05), while that in constipation-predominant IBS was significantly increased (P<0.05). At the same time, the results showed that compared with expression of claudin-1 mRNA in the colonic mucosa of the control group, claudin-1 mRNA expression in the diarrhea-predominant IBS group was significantly decreased (P<0.05), while claudin-1 expression in the constipation-predominant group was significantly increased (P<0.05). Values are expressed as the standard error of three samples. FQ-PCR, fluorescence quantitative polymerase chain reaction; IBS, irritable bowel syndrome.

Compared with claudin-1 mRNA expression in the small intestine as well as the colonic mucosa of the control group, it was significantly decreased in diarrhea-predominant IBS (P<0.05), while it was significantly increased in constipation-predominant IBS (P<0.05) (Fig. 3). Accordingly, there was a significant difference between the diarrhea-predominant group and the constipation-predominant group (P<0.05).

**Figure 4:**
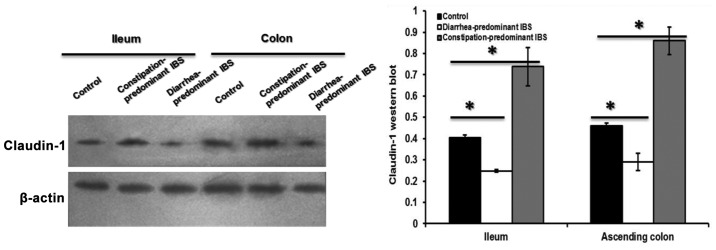
Western blot analysis of intestinal claudin-1 protein. Compared with the control group, claudin-1 protein levels in the small intestine and colon mucosa in constipation-predominant IBS were significantly increased (P<0.05), while the expression was decreased in the diarrhea-predominant IBS group (P<0.05). Values are expressed as the standard error of 3 samples. IBS, irritable bowel syndrome.

Compared with those in the control group, claudin-1 protein levels in the small intestine as well as in the colon mucosa in the constipation-predominant IBS group were significantly increased (P<0.05), while the expression was decreased in the diarrhea-predominant IBS (P<0.05) (Fig. 4). Changes in the protein levels of claudin were therefore in accordance with the changes in mRNA expression as determined by PCR.

### Immunohistochemical analysis of intestinal claudin-1 protein

**Figure 5:**
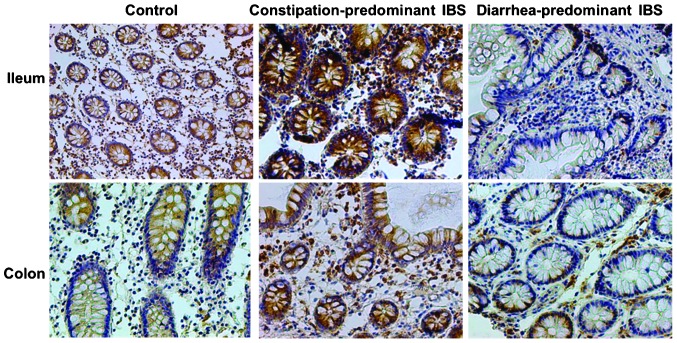
Immunohistochemical analysis of intestinal claudin-1 protein. Compared with expression in the small intestine and colonic mucosa in the control group, the expression of claudin-1 protein in the diarrhea-predominant group was significantly decreased (P<0.05), while that in the constipation-predominant group was significantly increased (P<0.05). However, no significant changes in the distribution of claudin-1 protein within the small intestine and colonic mucosa were detected among the groups, with location mainly in the cell membrane, the outer membrane and the cytoplasmic membrane. Positive staining for claudin-1 was indicated by a brown color, and no expression was present in the nuclei and nuclear membrane (magnification, ×200). IBS, irritable bowel syndrome.

Claudin-1 levels were determined based on the OD values calculated by the image analysis software. In analogy with the results of western blot and PCR analyses, it was found that, compared with the control group, expression of small intestinal as well as colonic mucosal claudin-1 protein in the diarrhea-predominant group was significantly decreased (P<0.05), while that in the constipation-predominant group was significantly increased (P<0.05), and the differences between the two IBS groups were significant (P<0.05), as shown in Table II and Fig. 5. Furthermore, no significant changes in the distribution of small intestinal as well as colonic mucosal claudin-1 protein in each group were found, with location mainly in the cell membrane, the outer membrane and the cytoplasmic membrane. Brown staining indicated positivity for claudin-1, and no expression in the nuclei and nuclear membrane was present (Fig. 5).

## Discussion

A previous study has reported that, among multiple molecular mechanisms, altered distribution of TJ protein expression as well as increased epithelial apoptosis affect intestinal permeability in patients with IBS (15). Furthermore, previous studies have indicated that the dysfunction of the intestinal epithelial barrier is associated with a significant disruption of occludin and claudin-1 expression (16–18).

The present study showed that intercellular TJ structures of the terminal ileum and ascending colon mucosa of constipation-predominant IBS patients were as normal as those in the control group, and no widened gaps or the tracer extravasation phenomenon were observed. In patients with diarrhea-predominant IBS, TJ gaps of the ileum mucosa and ascending colonic mucosa were widened, and varying degrees of tracer extravasation phenomenon were observed. Additionally, diarrhea-predominant IBS and constipation-predominant IBS had significantly increased mucus secretion and mucus bubble fusion in goblet cells of colon and small intestinal epithelium, which were in a state of exuberant secretion. Plasma cells in four patients with IBS and a history of infectious diarrhea were increased, the rough endoplasmic reticula of which were dilated and mitochondria were significantly increased. By contrast, structural changes of constipation-predominant IBS were not manifested. Neuroendocrine cells were observed in the two types of IBS patients, and the cytoplasm of neuroendocrine cells was filled with a high density of endocrine granules, several of which were vacuole-like, while in the normal control group, fewer neuroendocrine cells were observed in the small intestine and colon mucosa, the endocrine particles of which were fewer, and no significant changes in vacuoles were present. In addition, several vacuoles were fully filled with high-density particles in the mast cell cytoplasm in the two types of patients with IBS, which were in an active degranulation state, while a low density of particles and no vacuoles were present in mast cells of the colonic mucosa and small intestine of the normal controls.

Of note, Barbara *et al* (19) reported that the number of tryptase-positive cells in patients with IBS was three times that in normal individuals, and provided evidence of mast cell degranulation (19). Another study suggested that in patients with bowel symptoms, the number of endocrine cells in the colon increased at the in initial stage, while it was reduced in the chronic phase (20). These two studies are consistent with the results of the present studies.

In addition, ultrastructural analysis of samples from patients with IBS showed that goblet cells, mast cells, plasma cells and neuroendocrine cells had a highly active function and strong secretion, indicating that the nervous-immune-endocrine system may have an important role in the pathogenesis of IBS.

Mast cells have an important role in the innate immune system and are common residents of the intestine, contributing to the modulation of numerous pathophysiological processes in the gastrointestinal tract (21). Mast cells have been widely studied in IBS and results showed that their number was enhanced and that they were effectively activated, leading to the generation of visceral hypersensitivity and acute abdominal pain (22–24). Further studies reported the proximity of mast cells to enteric nerves in the colonic mucosa of IBS patients, which indicated that there was a communication between the central nervous system and the gastrointestinal tract (25,26). Therefore, intestinal function and inflammation are influenced by stress apart from other factors, including luminal bacteria. In fact, mast cells release inflammatory factors, including granulocytes, tumor necrosis factor alpha, interleukins 3–6, prostaglandins, platelet-activating factor, leukotriens, monocyte colony-stimulating factor and even certain specific proteases, such as carboxypeptidase-A and tryptase, to modulate paracellular permeability, which has a certain association with TJ proteins (27).

The expression and distribution of claudins is associated with TJ permeability. According to the results of the current study, compared with those in the control group, claudin-1 protein levels in the small intestine and colon mucosa in constipation-predominant IBS group were significantly increased (P<0.05), while expression was decreased in the diarrhea-predominant IBS group (P<0.05), the results of which were consistent among FQ-PCR, western blot and immunohistochemical analyses.

The results of the present study led to the conclusion that claudin-1 has an important role in changes in TJs in the intestinal mucosa in patients with IBS, which was not associated with the distributional changes of protein, but with TJ protein expression. The pathology of IBS patients with symptoms including changes in bowel movement traits and habits is not only associated with sensory and motoric disorders of the gut, but also with a variety of changes in intestinal TJ proteins. Increased expression of claudin-1 led to decreased intestinal TJ permeability, obstructing water and electrolyte leakage and resulting in constipation; on the contrary, reduced expression of claudin-1 leaded to enhanced intestinal TJ permeability, which increased penetration of water and electrolytes, resulting in diarrhea.

The present study investigated the molecular and cellular mechanisms of TJ dysfunction in IBS. Based on ultrastructural observation of intestinal TJs and epithelial cells, it was found that small intestine and colon samples of patients with constipation-predominant IBS and diarrhea-predominant IBS exhibited changes compared with those from normal controls, with changes of goblet cells, mast cells, plasma cells and neuroendocrine cells, which exhibited a highly active function and strong secretion. Furthermore, using various methods, it was found that claudin-1 protein levels in the small intestine and colon mucosa in constipation-predominant IBS were significantly increased compared with those in normal controls, while expression was decreased in samples from patients with diarrhea-predominant IBS.

In conclusion, the present study indicated that cellular changes as well as claudin-1 levels were associated with Tjs in IBS. Further studies are required to investigate the association between claudin-1 protein levels and different types of IBS.

## Figures and Tables

**Table I:** Primers used for polymerase chain reaction.

Gene	Upstream primer	Downstream primer	Length	Genbank number
GAPDH	TGAACGGGAAGCTCACTGG	TCCACCACCCTGTTGCTGTA	307	NM_008084
Claudin-1	ATGGTATGGCAATAGAATCGT	GCCTTGGTGTTGGGTAAGAG	179	NM_021101

**Table II:** Immunohistochemical analysis of intestinal claudin-1 protein (mean ± standard deviation).

Site	Group	Number	Claudin-1
Small intestine	Control	20	38.726±2.880
	Diarrhea-predominant group	23	31.376±1.971^a^,^b^
	Constipation-predominant group	20	45.084±2.230^a^
Colon	Control	20	37.459±3.343
	Diarrhea-predominant group	23	22.580±0.945^a^,^b^
	Constipation-predominant group	20	53.472±1.767^a^

a P<0.05 vs. control;

b P<0.05 vs. constipation-predominant group.
